# Systematic review on the impacts of agricultural interventions on food security and nutrition in complex humanitarian emergency settings

**DOI:** 10.1186/s40795-024-00864-8

**Published:** 2024-04-19

**Authors:** Melodie Al Daccache, Berthe Abi Zeid, Leila Hojeij, Ghassan Baliki, Tilman Brück, Hala Ghattas

**Affiliations:** 1 Center for Research on Population and Health, Faculty of Health Sciences, American University of Beirut, Beirut, Lebanon; 2 ISDC - International Security and Development Center, Berlin, Germany; 3 Zero Hunger Lab, Thaer-Institute, Humboldt-University of Berlin, Berlin, Germany; 4 Department of Health Promotion, Education, and Behavior, Arnold School of Public Health, University of South Carolina, Columbia, USA; 5 Leibniz Institute of Vegetable and Ornamental Crops (IGZ), Großbeeren, Germany; 6 Department of Nutrition and Food Sciences, Faculty of Agriculture and Food Sciences, American University of Beirut, Beirut, Lebanon

**Keywords:** Systematic review, Nutrition-sensitive, Agriculture, Food security, Nutrition, Health, Complex and humanitarian emergency settings

## Abstract

Complex humanitarian emergencies are a main driver of food and nutritional insecurity. Agricultural interventions are key to improving nutrition and food security, and their positive impacts are well-documented in stable developing countries. However, it is unclear if their positive effects on food security hold in complex emergency settings, too. In this paper, we systematically review empirical articles that apply rigorous designs to assess the causal impacts of agricultural interventions on food security, nutrition, or health outcomes in complex humanitarian emergencies. We only find six articles matching these criteria, which have mixed results on dietary diversity and food security, and little evidence on child nutrition. Our review underscores the need for more rigorous research on the impacts of agricultural interventions in complex humanitarian emergency settings.

## Introduction

Complex humanitarian emergencies involve violence (including political, economic, military and social violence) and are characterized by disease, hunger, and displacement [1, 2]. They are becoming increasingly severe and protracted, having caused the displacement of around 110 million people around the world in 2023 [3]. Complex humanitarian emergencies damage economic and social assets, limit access to land and water, destroy rural infrastructure and weaken markets, all of which have a detrimental impact on food production, consumption, and distribution [4]. Households in complex humanitarian emergency settings (CHES) thus have limited access to safe, affordable and nutritious food, a situation which is often compounded with a lack of access to clean water, essential health services, and optimal feeding practices [5]. Complex humanitarian emergencies, along with climate stresses, are hence major drivers of food insecurity and hunger [6, 7]. In fact, violent conflict has been identified as the most consistent predictor of under-5-year-old child malnutrition, with 80% of the world’s stunted children living in countries affected by violent conflict [8]. Wars have far-reaching repercussions on agriculture along the supply chain, leading to deterioration of agricultural assets, irrigation systems, and infrastructure and reducing food production, agricultural growth, and worsening rural livelihoods [6, 9–10].

Agricultural and small-holder interventions targeting small-scale livestock, fish, crop or horticultural production have been flagged as a crucial tool to combat hunger to meet the 2030 Agenda for Sustainable Development 2 (SDG2) [11], particularly due to their potential in improving income generation, purchasing power, dietary diversity and nutritional quality [12–19]. In the past decade, such interventions have increasingly been implemented in humanitarian and conflict-affected settings and are hypothesized to be key for building resilience and overcoming food insecurity [4, 20].

There is growing evidence for nutrition-sensitive agricultural interventions in stable developing settings. A number of systematic reviews in the past decade have investigated the impacts of agricultural intervention as an integral component of improving food security and health in Low- and Middle-Income Countries (LMICs) [21–25]. These reviews included a wide range of studies analyzing different types of agricultural support such as homestead food production, home vegetable gardens, biofortification, livestock and fisheries, dairy, and irrigation programs. Their findings were consistent in showing positive impacts of agricultural support on household production of agricultural goods, dietary diversity, and income [23, 24]. The most recent systematic review demonstrates the effects of nutrition-sensitive agricultural interventions on nutrition and health outcomes, especially for women and children [25]. Moreover, agricultural interventions were found to facilitate women’s contribution to household food availability and accessibility and to moderately increase children’s consumption of food rich in protein, vitamin A, and micronutrients [23, 24]. Investigating the role of agricultural support on women’s empowerment along the causal pathways from agriculture to nutrition, Ruel et al. identified an improvement in specific dimensions of women’s empowerment including social capital, ownership, and decision-making [25]. However, there is no evidence for significant positive impact on downstream health outcomes such as child stunting, wasting, and underweight [21, 23, 25].

However, given that exposure to complex humanitarian emergencies shapes economic decision-making [26], production, marketing and consumption behavior [4, 20], and access to land and water [27], theorized and tested mechanisms from stable developing settings might not hold, or be relevant, in CHES. For example, in CHES, farmers may have restricted access to land and water resources potentially constraining them from taking up the interventions. Even when farmers access land and water, CHES can lead to loss of productive and livestock assets, crop damage, and agricultural labor shortages, which leads to low harvests [4]. At the same time, CHES limit access to output and value chain markets for selling agricultural produce, constraining income-generation and reducing the availability and supply of fresh produce in markets [4].

However, only one study included in the previous reviews was conducted in a setting affected by a complex humanitarian emergency [28]. Considering that a large part of the global burden of food insecurity, hunger and poor nutritional status occurs in such contexts, it is important to generate and compile evidence on what works in CHES if SDG2 is to be met. Additionally, because of the complexities of intervention design and implementation in CHES, lessons on whether and how agricultural interventions reduce hunger from stable settings cannot be generalizable or transferable [4].

To address this gap in the literature, the goal of this systematic review is to compile, summarize, and assess the rigor of existing evidence on the impact of nutrition-sensitive agricultural interventions on food security and nutrition outcomes in CHES. Any peer-reviewed journal articles or published reports identified through the search databases between the years 1980 and 2022, conducted in CHES with at least one type of agricultural intervention, with a comparator group and a focus on nutrition, health, or food security outcomes were included in the study.

## Methodology

### Study design and search strategy

In this systematic review, we define CHES to include those experiencing active armed violence or protracted episodes of violent conflict which lead to humanitarian emergency and forcible displacement. This also includes post-conflict settings, where active violence subsided but countries remain at high risk of relapse and the repercussions of the violence remain prevalent. We define nutrition-sensitive agricultural interventions as any program in the primary sector that addresses the underlying causes of food insecurity and/or malnutrition such as biofortification, homestead production, livestock and dairy, agricultural extension, irrigation, aquaculture, and value chains.

We start by identifying four key systematic reviews [23–25, 29] and an overview of reviews [22] published since 2012 that focused on the impacts of agricultural intervention on food security and nutrition. We hand-searched the references in these reviews and identified 160 references to be screened for inclusion according to our definitions of complex humanitarian emergency settings. We then replicated the search of the most recent study conducted by Ruel et al. [25], which summarized key findings from studies focusing on the nutritional impact of agricultural programs. This review helped formulate the search strategy and identify the types of agricultural interventions to include in our review. To include all our search terms of interest, we added keywords on food security outcomes, conflict, and complex emergency settings. We then ran this search covering publications from 2017 onwards to identify studies that focus on agricultural intervention and food security, nutrition, and health in populations affected by conflict and humanitarian emergency.

Search terms for nutrition-sensitive agricultural interventions are included in Table 1 and partially derived from [22] and [25] to ensure that the results are comparable. We used standard systematic review guidelines, as outlined in the Preferred Reporting Items for Systematic Reviews and Meta-Analysis (PRISMA) statement [30]. The list of search terms used to identify articles for the review are presented in Table 1. The protocol was registered in PROSPERO under CRD42022327049.

**Table 1:** Search topics and terms used in the review of nutrition-sensitive agricultural programs on food security in populations affected by humanitarian crises

Topic	Search terms
Outcomes	
Nutrition and food security	“nutrition* outcome*” OR “nutrition* status” OR “diet* diversi*” OR micronutrient* OR anthropom* OR food* OR macronutrient* OR nutrition* OR “food consumption*” OR diet* OR “food secur*” OR “food insecur*”
Health	health* OR morbidit* OR mortalit* OR prevalence* OR incidence* OR burden* OR disease* OR “health status*” OR “health outcome*”
Interventions	
Biofortification	biofortif* OR bio-fortif* OR “harvestplus” OR “harvest plus”
Homestead production	“homestead production” OR “homestead food production” OR “home garden*” OR “homestead garden” OR “vegetable garden*”
Livestock and dairy	(“livestock program*” OR “livestock production*” OR “livestock ownership” OR “dairy production” OR “dairy program” OR “dairy development” OR “animal husbandry” OR “poultry development” OR “poultry production” OR “poultry program” OR “organic farming” OR “livestock intervention*”) AND agriculture
Agriculture extension	“agricultur* extension” OR “agricultural commercialization” OR “horticulture”
Irrigation	(Irrigation OR “water management”) AND impact
Aquaculture	(Aquaculture OR fisheries or fishpond) AND agriculture
Value chains	“value chain” OR value-chain OR “value crop*” OR “value-crop*” AND (nutrition OR diet)
Nutrition-sensitive agriculture	(“nutrition-sensitive” OR “nutrition sensitive”) AND agriculture
Interventions	(program* OR polic* OR strateg* OR legislation* OR law* OR intervention* OR technique* OR planning OR practice* OR fiscal OR regulation* OR sustainable OR tax* OR subsid* OR procurement* OR incentive*) AND (agriculture)
Contexts	
Conflict	(Conflict* OR disaster* OR war* OR shock* OR humanitarian* OR emergenc* OR catastrophe* OR crisis OR crises OR violence) NOT “conflict of interest”
Refugees and migrants	refugee* OR UNHCR OR displace* OR “forced migrant*” OR “forced migration*” OR “forced displacement” OR “forcibly displaced”

For each database search, we used Boolean operators “AND” to pair the search terms of the outcomes section with the search terms of the context section with the search term of each type of intervention as listed in Table 1. The operator “OR” was used for different synonyms of the same topic (for example, conflict OR crises OR emergency). The “OR” was used to expand our outcomes search by adding all the relevant keywords of nutrition, health, and food security. The same approach was used to add search terms related to complex humanitarian emergencies. The “NOT” operator was used to exclude studies that only identified “conflict of interest” without any specific conflict-related search term in the text.

We systematically searched published studies in the following databases: Scopus, PubMed, and Web of Science. The search was carried out on 28 March 2022, restricted to peer-reviewed and impact evaluation articles published in English language, and conducted in populations affected by CHES from 2017 onwards. Animal studies were excluded from this review. The number of articles identified through the first stage are reported in Table 2, by topic and databases. The search strategy was first piloted in Scopus on 5 March 2022. Given the considerable number of studies included in the search results, the key terms for the outcomes (e.g., health, food security, and nutrition) and those for the context (e.g., conflict and refugees, and migrants) as well as the intervention topic (e.g., program and policy) were restricted to title, abstract, and keywords search. To ensure the inclusion of all studies that discussed at least one type of agricultural program, each intervention type was searched for all fields in the three databases. The same search strategy was replicated in the other databases and the results are shown in Table 2.

In order to identify and capture unpublished relevant reports, we conducted a broad search on Google Scholar for the impacts of agricultural intervention on food security and nutrition in populations affected by CHES. The first 60 studies identified were exported and added to the screening stage. We also searched ReliefWeb and filtered for ‘evaluation and lesson learned’, yielding an additional 22 results. ReliefWeb was used given the focus of the database on global crises and disaster-affected settings [31]. A parallel search was also conducted on the International Initiative for Impact Evaluation (3ie) database to further identify high-quality impact evaluation studies conducted in CHES.

**Table 2:** Number of articles identified by the type of agricultural program and database

Type of agricultural programs	Scopus (9,263)	PubMed (371)	Web of Science (696)
Biofortification	249	7	25
Homestead food production system	237	3	13
Irrigation	3,395	40	51
Agricultural extension	1,435	36	34
Livestock and dairy	1,415	87	69
Aquaculture	1,848	76	170
Value chain	446	2	54
Nutrition-sensitive agriculture	93	4	6
Intervention	3,124	159	366

### Eligibility criteria

We used eight criteria to determine eligibility for inclusion of full-text review: Any peer-reviewed journal article or published report written in the English language between the years 1980 and 2022 and conducted in CHES with at least one type of agricultural intervention, with a focus on nutrition, health, or food security outcomes were included. The review was limited to studies with a comparator, either between intervention and control groups or differences between pre- and post-intervention in the same group. Excluded studies included systematic or scoping reviews, literature review, or any study that did not use agricultural support for the sake of improving nutrition, food security, or health outcomes. Studies that implemented a program with agricultural support being one of its intervention components, were excluded from the review if the analysis did not assess the impact of agricultural support alone on the selected outcomes.

We defined studies as conducted in CHES if they met the following criteria: (1) the study was conducted in a country ranked among the 10 countries with the lowest political stability as measured by the political stability index (which measures “perceptions of the likelihood that the government will be destabilized or overthrown by unconstitutional or violent means, including politically-motivated violence and terrorism”) [32], and the intervention took place after the onset of the crisis as measured by this, or the country in which the study was conducted had an active humanitarian response from UNOCHA at the time of the intervention; and (2) the authors explicitly mentioned that the study was conducted in CHES, or had recently experienced episodes of complex humanitarian emergency and was still affected by the consequences of the crisis.

Included articles are classified into the following two categories: population living in (post-) conflict and emergency settings and populations living in protracted conflict settings. A detailed plan for inclusion and exclusion criteria is described in Table 3.

**Table 3:** Inclusion and exclusion criteria for the full-text review of nutrition-sensitive interventions

Criteria	Inclusion	Exclusion
Publication type	- Peer reviewed - Published papers and reports - Grey literature (working papers)	- Evidence/policy brief, conference - Unpublished abstract, study protocol - Meta-analysis, systematic or scoping review
Publication year	1980–2022	< 1980
Language	English	Others
Study type	- Qualitative, quantitative, or mix-method design - Impact evaluation	- Literature review - Feasibility study
Intervention	Any agriculture intervention used as a livelihood strategy for food or income of the household such as: - Biofortification or harvest plus - Homestead production or vegetable garden - Irrigation or water management - Value chain/crop - Livestock and dairy - Agriculture extension or horticulture	Any agriculture intervention not used as a livelihood strategy (e.g., leisure activity not intended for food or income of the household)
Comparator	-Studies comparing outcomes between different groups or difference before and after the intervention of the same group - Cross-sectional studies comparing beneficiaries with non-beneficiaries	- No comparator/control group
Outcomes	- Food security - Health/disease - Diet and diet diversity - Micronutrient/macronutrient intake or status - Nutrition status/outcomes - Anthropometry	- Nutrition awareness, perception, attitudes - Food safety
Settings	- Countries classified with a high political instability index, or - Country received an active humanitarian response from UN OCHA at the time of the intervention, and - The authors explicitly mentioned that the study was conducted in CHES, or had recently experienced episodes of violent conflict (refer to Table 1 for the full list of context-related search terms) and was still affected by the consequences of the crisis	Stable or non-humanitarian (including LMIC who did not experience conflict or humanitarian crises)

### Selection process

The results found from our search were downloaded into the reference management EndNote X9 software and duplicate records were removed. The remaining studies were imported to Covidence software for title and abstract screening according to the inclusion/exclusion criteria as described in Table 3. First, the screening process trialed by MD and LH on 150 articles during a preliminary search yielded the inclusion of 2 articles and revealed a high interrater agreement. Then, the same authors, in addition to a third reviewer BZ screened the remaining articles, and the papers selected for full-text review were retrieved for further examination. The inclusion criteria were applied against these papers independently by three reviewers (MD, LH, BZ). Disagreements were resolved by discussion and consensus with HG.

Finally, information was extracted from the eligible studies including author and title, year of publication, country and region (if available) of the intervention, type of crises, type of agricultural intervention, outcome indicators, study method, type of design, summary of the findings related to nutrition, food security, and health. We also extracted data on intermediate outcomes (agricultural productivity, assets and income) to clarify potential impact pathways. Data were also extracted on other adverse or unexpected findings, author’s recommendations and limitations, and conclusion of the study.

### Assessment of risk of bias

The Cochrane Risk of Bias In Non-randomized Studies of Interventions (ROBINS-I) tool was used by two independent reviewers (MD and BZ) to assess the risk of bias [33]. Any disagreement in quality assessment of these studies were resolved by consensus discussion with HG.

## Results

**Fig. 1:**
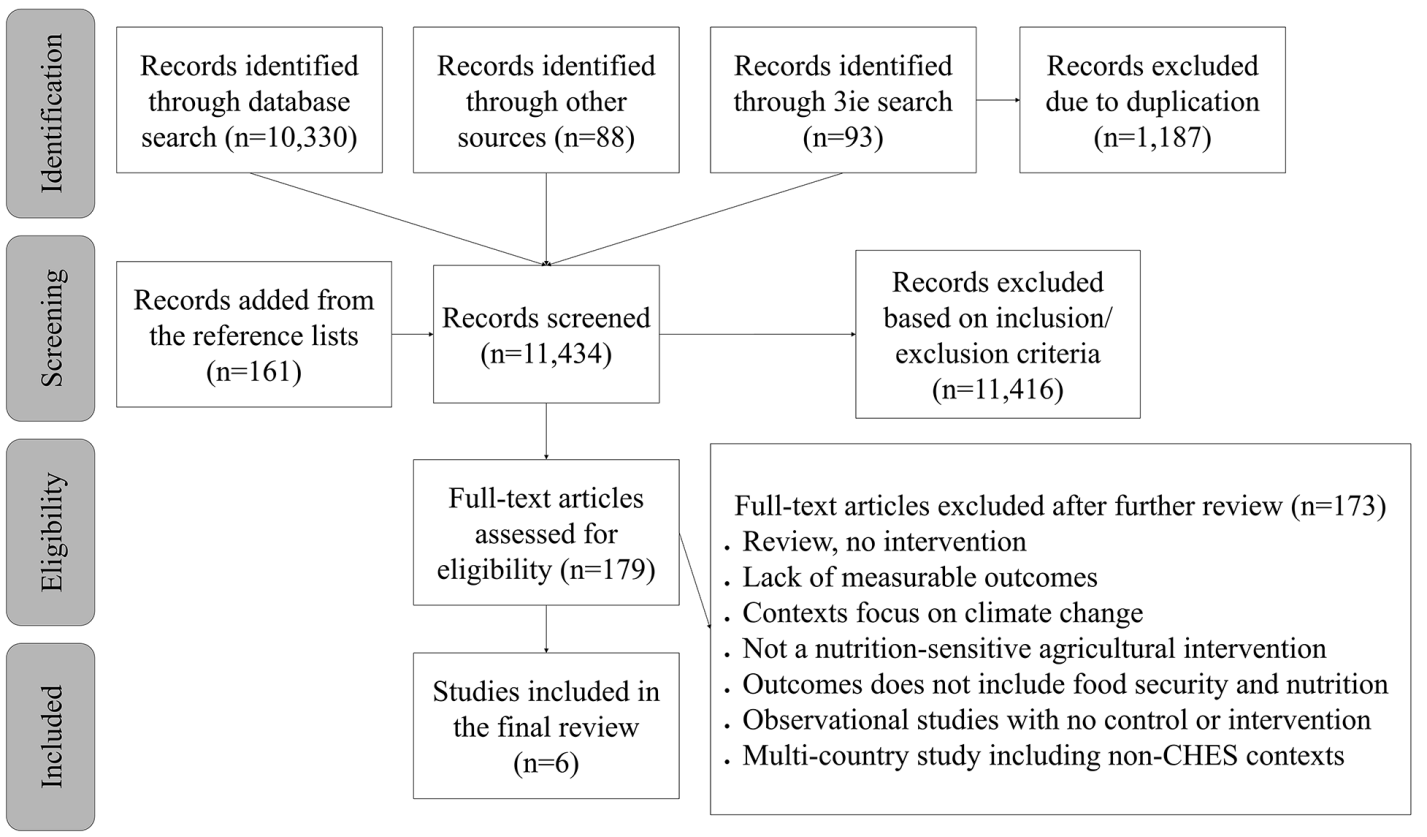
Search strategy flow diagram

A total number of 10,511 articles were identified in the first round of search in which 10,330 articles were identified through search databases, 88 articles from ReliefWeb and Google Scholar, and 93 articles from 3ie. Using EndNote, 1,187 duplicate records were excluded, and the remaining 11,434 articles were screened for title and abstract using the inclusion and exclusion criteria specified in Table 3. We added to the screening phase an additional 161 articles identified from the reference list of our key systematic reviews. A total of 179 articles were screened for full-text, and after reading carefully, 173 articles were excluded because they did not meet the eligibility criteria (reasons underlined in Fig. 1). For example, studies limited to agricultural production as an outcome without assessing food security and nutrition outcomes were excluded. Observational studies that did not include a comparator group or an agricultural intervention were also excluded from this review. Only 6 articles were identified to meet our eligibility criteria and were proceeded to data extraction. Our review was limited to peer-reviewed articles, working papers, and published reports.

**Table 4:** Data extraction of the included articles

Authors, year, study location	Type of intervention	Evaluation design	Outcomes measured	Findings	Conclusion
***Population living in (post-)conflict settings***					
Doocy et al., 2019 [34] Eastern Democratic Republic of Congo Evaluating interventions to improve child nutrition in Eastern Democratic Republic of Congo	**Jenga Jamaa II includes**: - Income generation though FFS and F2F: training on agricultural methods, provision of seeds and tools, and farmers train other members in their community - Improve health and nutritional status of children < 5 years through PM2A: messages on child health, nutrition education and behavioral change, promote homegardens, monthly ratios, and health system support - Empower FI women through WEG: meetings to deliver literacy, numeracy, business, marketing training, and the provision of goats and kits	- Community-matched quasi-experimental design - Communities received one intervention versus multiple interventions versus no intervention - Program implemented between 2011 and 2016 − 1312 children from 1113 HH participated - Surveys 3.5 years apart	**Children’s outcome measures**: - DDS measured using 24 h recall - Minimum dietary diversity achieved if child consume ≥ 4 food groups - Minimum acceptable diet met if child achieved both minimum meal frequency and dietary diversity - Stunting - Underweight	**Children’s food security**: - Modest improvement in DD for PM2A and FFS interventions compared to control group - Increase in the minimum DDS in PM2A and FFS groups compared to the control group - Minimum meal frequency was met for the PM2A group as compared to the control group **Children’s nutrition**: - No significant difference for stunting or underweight - Modest decrease in the prevalence of underweight among PM2A group and stunting among PM2A and FFS groups	PM2A and FFS groups yielded better child dietary measures and nutrition outcomes, particularly among the intervention with a behavioral change component (PM2A)
Doocy et al., 2018 [35] Eastern Democratic Republic of Congo Improving household food security in eastern Democratic Republic of the Congo: a comparative analysis of four interventions	Same as above	Same as above but without considering children’s sample	**Household (HH) outcome indicators**: - HDDS measured over the past 24 h - Target dietary diversity achieved if HH consume ≥ 5 food groups - HFIAS	**HH food security indicators**: - Significant increase in HDDS for those who received WEG, PM2A, or FFS - Significantly lower HFIAS score for WEG, PM2A, and FFS interventions, with smaller gain in F2F - Pathway: WEG, PM2A indirectly improved food security through income generating activities and HH gardens	WEG, PM2A, and more specifically FFS interventions significantly improve HDDS and HFIAS, a lower impact was observed for F2F intervention
Doocy et al., 2017 [36] Eastern Democratic Republic of Congo Food security and nutrition of farmer field schools in Eastern Democratic Republic of the Congo	- Same as above focusing on FFS program (one component of the Jenga Jamaa II project) - Combined with qualitative data (KIIs, FGD) after the end of the project	- Same as above - FFS beneficiary and controls were selected while the program was operating. − 388 beneficiaries and 324 controls were enrolled	**Household outcome indicators**: - HDDS - HFIAS **Children’s outcome indicators**: - Stunting - Underweight	**Agricultural production techniques**: - FFS increased the number of agricultural techniques, more specifically for weeding, hoeing, and row planting **Use of marketing and financial services**: - More HH used joint negotiation, farmer business association levels, and sales through agricultural collection centers. - Use of informal credit significantly decreased and use of savings increased **HH food security**: - HDDS and HFIAS significantly improved in FFS **Children’s nutrition**: - No significant difference in the prevalence of child stunting and underweight	- This program diversified agricultural production, improved HDDS and HFIAS. However, the nutritional status of children did not improve - Increases in agricultural production alone are not enough to induce change in child’s nutrition
***Populations living in protracted crises and displacement***					
Vallet et al., 2021 [39] South Sudan Where are the development actors in protracted crises? Refugee livelihood and food security outcomes in South Sudan demonstrates the potential for fragile settings	- UNHCR livelihood intervention includes: - Agriculture: inputs and agricultural training - Small business: vocational training and business support - VSLA - Complemented with qualitative data (FGD, KIIs)	- Mixed methods approach - RCTs - Program implemented between 2016 and 2018 - HH received livelihood training package alone (agriculture, small business development, or other types of trainings alone or in combination) versus the same training plus VSLA - Qualitative data collected at the end	**Household outcome indicators:** - FCS	**HH assets, income, access to market and financial services:** - VSLA plus training has a significant impact on HH productive assets, income source, access to markets, and financial services as compared to one type of training only **Food security, coping strategies and recovery from shocks: ** - Significant increase in food security, ability to meet food and non-food needs and recover from shocks for those who received livelihood plus compared to training only or other types of training only ** Other outcomes with potential health implications (qualitative work):** - Livelihood program increased social cohesion by reducing refugee- host community conflict - Livelihood program decreased sexual and gender-based violence	- UNHCR program improved food security, livelihood, and income-generation in volatile and unsecure settings. - The outcomes were much improved when the training was complemented with VSLA
Baliki et al., 2018 [37] North-East Nigeria Drivers of resilience and food security in North-East Nigeria: Learning from the Micro Data in an Emergency Setting	FAO program includes the provision of quality agricultural inputs such as cereals, pulse and vegetable kits	- Quasi-experimental design with repeated cross-sectional surveys - Data collected from 5,807 HH at baseline and 5,991 HH at endline - Beneficiaries (intervention group) were compared to non-beneficiaries (control group with no intervention)	**Household outcome indicators:** - FCS - RCSI - Resilience measured by the use of harmful livelihood strategies over the past 30 days	**Food security indicators:** - FCS improved significantly for the beneficiary group, particularly among IDPs and those residing in high and extreme conflict-affected areas. - RCSI significantly increased among the beneficiary group, particularly among HH residing in low conflict areas. - The program builds HH resilience, except for those who experienced a personal shock **Other outcomes with potential health implications:** - Intervention improved social cohesion by mitigating participant’s concern about conflict between community members and local security	The provision of agricultural inputs increased FCS shortly after the intervention, and are likely to builds resilience to shocks, especially among the most vulnerable
Leuveld et al., 2018 [38] Eastern Democratic Republic of Congo Agricultural extension and input subsidies to reduce food insecurity. Evidence from a field experiment in the Congo	N2Africa includes agriculture extension intervention and input subsidy programme	- Clustered-randomized experimental design - Compared villages who received extension program alone versus extension program + subsidy scheme - Program implemented in 2013 − 265 HH received training only and 256 HH received training with subsidy	**Household outcome indicators:** - Yields (kg/hectare) - HFIA	**Use of agricultural inputs:** - Fertilizer and inoculant uptake significantly increased in villages who received training + input subsidy compared to villages who received training only ** Food production:** - No significant impact on beans and cassava yields ** Food insecurity:** - No significant impact on food security outcome ** Market access:** - Villages with low proximity to markets have lower use of agricultural inputs	-The intervention was successful in increasing the use of yields enhancing inputs: a new technology called inoculant and chemical fertilizers - The increase in adoption of agricultural input did not translate to better yield or food security

Table 4 presents the characteristics of the articles included in this review, by type of humanitarian setting. Studies were either conducted in areas affected by conflict or hosting displaced populations who fled complex humanitarian emergencies. A total of six articles (or four studies) were identified, from which three articles were conducted post-conflict and three articles in protracted humanitarian crises.

### Studies conducted in (post-) conflict settings

Three articles resulting from the Jenga Jamaa II project on food security and child nutrition outcomes in two territories severely affected by previous conflict in the Democratic Republic of Congo (DRC) were reviewed [34–36]. This community-matched quasi-experimental study aimed at increasing income of food insecure farmers though (1) farmers field school (FFS) and farmer-to-farmer (F2F) interventions, (2) the prevention of malnutrition in children under two approaches (PM2A); i.e., the promotion of home gardens complemented with a behavior change component to support young child nutrition, and (3) empowering food insecure women through women’s empowerment groups (WEG). The Jenga Jamaa II project was implemented by the Adventist Development and Relief Agency in South Kivu between the years 2011 and 2016.

The first paper looked at the impact of FFS on food security and children’s anthropometry in post-conflict Eastern DRC [36]. The FFS intervention provided experience-based education on farming practices, crop handling, entrepreneurship skills, and delivered seeds and tools packages to farmers. Compared to the control group who did not receive any intervention, the beneficiary group that received the four-year FFS program had improvements in agricultural production techniques, such as weeding (96.2%), hoeing (95.9%), and row planting (92.7%) practices, the adoption of several marketing strategies including the use of joint negotiation (68.8%), and farmer business association levels (56.3%). Using propensity score weights to balance on baseline characteristics of the intervention and control groups, the intervention was found to significantly improve food security outcomes, including an increase in Household Dietary Diversity Score (HDDS) (+ 0.9 points) and a decrease in Household Food Insecurity Access Scale (HFIAS) (-4.6 points), but had no impact on child nutritional status such as stunting and underweight. Despite these reported benefits, the authors acknowledged that the impact pathways through which the agricultural intervention affected food consumption was not fully understood mainly due to poor data quality on agricultural outputs and yields.

The second article from the same program focused on multiple intervention components and showed that PM2A and WEG had similar positive results to the FFS component [35]. While the F2F intervention did not improve HDDS among the beneficiary group, a modest non-significant decrease in HFIAS was reported. However, despite the use of propensity scores to account for observable characteristics of a non-randomized design, selection bias relating to the willingness of farmers to participate in the intervention could have affected the results. Similarly to the previous article published by Doocy [36], this study was unable to demonstrate the mechanism underlying the improvement in food security outcomes and suggested that further research be conducted in this post-conflict setting.

The third article looked at the same Jenga Jamaa II project’s components focusing on children’s dietary diversity and nutrition [34]. Minimum dietary diversity among children was achieved for PM2A and FFS groups and only the PM2A group met the minimum meal frequency and acceptable diet targets, suggesting the importance of integrating a behavioral change component on children’s diet and feeding practice as part of PM2A. However, the improvement in children’s dietary intake was not necessarily translated into better nutrition, mainly due to the lack of precision in estimating birth dates to assess anthropometric data, and the low sample size which underpowered the study to detect changes in nutrition outcomes. Hence, this study recommended the need for future multi-component interventions targeting nutrition education, health, agricultural provision, and income generation to improve child diet and nutrition.

### Studies conducted in protracted humanitarian settings (with war refugees)

Three articles reported on studies conducted in protracted humanitarian crises. The first involved the assessment of an agricultural extension program that provides high quality agricultural inputs to internally displaced persons, returnees, and host communities, on food security and resilience indicators in North-East Nigeria using a repeated cross-sectional survey [37]. The program was implemented by the Food and Agriculture Organization (FAO) in 2017, and offered vegetables, cereals, and pulses kits to beneficiaries, and compared the changes in outcomes from baseline to endline. This study also compared the changes between beneficiaries and non-beneficiaries before and after the intervention to quantify the program’s mean impact on food security outcomes (using a difference in difference analysis). The results showed a significant improvement in the Food Consumption Score (FCS) (+ 5.4 points) in the beneficiary group as compared to the non-beneficiary group, with a particular increase among the Internally Displaced Populations (IDPs) and those residing in extreme conflict areas. In turn, the Reduced Coping Strategy index (RCSI) also significantly decreased among the beneficiary group (-0.9 point), particularly among those living in low conflict areas.

Findings from a working paper series undertaken by Leuveld et al., [38] implemented the N2Africa programme and targeted smallholder farmers in South Kivu, the Democratic Republic of Congo, a province undergoing protracted violent conflict with constant exposure to adverse climatic conditions [38]. The program aimed at improving agricultural yields, food security, and income through the delivery and dissemination of advanced technology. This program, which was implemented in 2009, collaborated with local organizations including six local Non-Governmental Organizations (NGOs) who had prior experience in implementing agricultural development initiatives. The N2Africa intervention encompassed agricultural extension services and an input subsidy program, where lead farmers with extensive experience in farming were selected from the community to work in a group of 15–30 farmers. All lead farmers received legume technology packages that included agricultural inputs for legumes of choice such as seed, fertilizer, and inoculant, among others. This program, in addition, provided training on plant spacing practices, education information on the nutritional benefits of legume consumption, as well as training on value-added processing of legumes to provide income opportunities specifically to women. Using a clustered-randomized design, villages were randomly selected to receive subsidy schemes with extension programs versus extension programs alone. Results showed that fertilizer and inoculant uptake significantly increased in villages that received the training with input subsidy compared to villages that received the training only. Using heterogeneous analysis, the study showed that villages with low proximity to market generally have low use of agricultural inputs, mainly due to increased cost of access. However, the increase in input use did not necessarily translate into better yields and food security, due to small sample size and low absolute use of agricultural inputs, limiting the study’s power to detect an impact on input use and nutritional outcomes. The authors suggested the need for larger interventions that target changes in market structure to develop local supply chains and improve market access to agricultural inputs by lowering their costs. The paper also highlights the challenging conditions in which the program took place and questioned program fidelity and the ability to correctly track households who received input packages.

In South Sudan, a challenging and fragile context with refugees living in an ongoing protracted crisis, the United Nations High Commissioner for Refugees (UNHCR) livelihood project implemented between 2016 and 2018 included two main interventions delivered to refugees in Maban and Unity refugee hosting areas: (1) an agriculture intervention that included training and inputs and (2) a business intervention that included vocational training and business support [39]. Using a randomized design, this project sought to compare those who received any livelihood intervention to those who received the same support plus increasing access to informal financial services through Village Saving and Loan Associations (VSLA). The results showed that household assets and income, access to markets and financial services, as well as food security, coping strategies, and recovery from shocks all significantly improved for refugees who participated in VSLAs combined with livelihood training as compared to agricultural training, business training, or other trainings (combination of skill training) alone. Qualitative work supported the conclusion that an integrated multi-component livelihood intervention improved household food security and nutrition outcomes, decreased credit use, increased savings, increased production and income generation. This in turn, increased households’ engagement with local markets, improved their ability to cope with shocks, and alleviated tensions that existed between communities. However, more respondents were concerned about theft and lack of safe places to hold savings due to the absence of formal financial institutions, suggesting the urgent need for aid actors to expand refugee’s economic inclusion in protracted crises.

Overall, we identified six eligible studies, from which three were conducted in post-conflict settings, and the remaining three were conducted in protracted humanitarian settings. All these studies implemented multi-component agricultural interventions, targeting vulnerable groups such as smallholder farmers, refugees, IDPs, returnees and host communities, including children. Five of these studies used quasi-experimental designs with no *‘pure’* control group. In addition, they highlighted that the impact pathways through which agricultural interventions affected food consumption were not fully understood, and called for further research to address this gap. Suggestions included incorporating nutrition training, targeting market structure and access, and lowering agricultural input costs.

Table 5 summarizes the outcome of the risk of bias assessment within the articles. Five articles were identified as having an overall moderate risk of bias [34–38]. Only one article was additionally identified as being at serious risk of bias mainly due to confounding [34]. A high degree of risk was mainly associated with bias due to confounding.

**Table 5:** Risk of bias assessment ROBINS-I tool

Risk of bias domains	Doocy 2019 [34]	Doocy 2018 [35]	Doocy 2017 [36]	Baliki 2018 [37]	Leuveld 2018 [38]	Vallet 2021 [39]
Bias due to confounding	Moderate	Moderate	Moderate	Moderate	Moderate	Serious
Bias in selection of participants into the study	Low	Low	Low	Moderate	Low	Moderate
Bias in classification of the interventions	Low	Low	Low	Low	Low	Moderate
Bias due to deviations from intended interventions	Moderate	Moderate	Moderate	NA	Moderate	NA
Bias due to missing data	Moderate	Moderate	Moderate	Low	Moderate	Low
Bias in measurement of outcomes	Moderate	Moderate	Moderate	Moderate	Moderate	Moderate
Bias in selection of the reported result	Low	Moderate	Low	Moderate	Low	Moderate
**Overall risk**	**Moderate**	**Moderate**	**Moderate**	**Moderate**	**Moderate**	**Serious**

## Discussion

Our review of evidence unveiled only six articles published that assessed the effectiveness of agricultural interventions on food security and nutrition in CHES, and none published before 2017. Clearly, this is a relatively low number of articles identified as compared to the increasing number of countries in need of humanitarian assistance and/or experiencing high political instability [32, 40]. Additionally, the geographic coverage of these studies was limited to Africa (one study in South Sudan and North-East Nigeria, and two studies in DRC), and none were from the Middle East, Asia, or South America.

Of these six articles, four were peer-reviewed and two were published reports, as compared with a relatively high number of peer-reviewed articles published from stable settings [23–25]. Our review applied stringent criteria for inclusion of studies and did not include observational designs that previous reviews considered which could explain the low numbers of studies identified. This indicates that although experimental and quasi-experimental designs are possible to implement in CHES, very little rigorous research linking agricultural interventions to food security and nutrition has been conducted in such settings, and the majority of studies were conducted in prolonged relief or recovery (protracted crises and post-conflict) rather than acute phases, highlighting a major research gap.

Homestead food production, agricultural extension, and livestock support alone or in combination were the only nutrition-sensitive agricultural interventions identified in CHES. These interventions were also common in stable settings, but the latter also often included development-oriented interventions such as biofortification, irrigation, and value chain support, alone or in combination to food production interventions. It is likely that agricultural input provision is the main agricultural intervention type implemented in CHES as it provides tangible assets to households, it is easy to distribute, and it generates immediate socio-economic and nutritional benefits. These interventions enable vulnerable households to establish and profit from small-scale local agricultural production during a crisis to improve their food security. Local production in CHES is essential to ensure adequate food supply particularly that the agricultural sector deteriorates significantly during complex humanitarian emergency periods [8].

The reviewed studies included the primary outcome indicators: food security, nutrition, and health. In addition we considered outcomes on the impact pathway: agricultural production, asset ownership, and income. Overall, the interventions showed a positive impact on the use of agricultural input and techniques, but no impact on agriculture production and yields [36–38]. Agricultural interventions increased income and savings and decreased the need to rely on credit but resulted in mixed evidence in regard to its impact in the sale of productive assets to deal with income shocks [36, 39]. The majority of the interventions demonstrated a positive effect on household dietary diversity and food security, yet one study did not demonstrate any significant impact [38].

The studies also report a modest increase in children’s dietary diversity, yet only two articles investigated the impact on prevalence of stunting and underweight among children, where none find any detectable significant impact [34, 36]. In fact, Doocy et al. [34] finds that incorporating a behavior change communication (BCC) component led to an increase in children’s minimum diet diversity and minimum meal frequency, consistent with a recent meta-analysis that finds a positive impact of nutrition-sensitive agriculture on diet diversity in children in stable settings, that is augmented when interventions include BCC [41].

Our results are largely consistent with findings from previous reviews conducted on studies in stable developing settings, which demonstrate a positive effect on the use of agricultural inputs and practices, and some mixed evidence on food production, consumption, and dietary diversity [16, 17, 21–25, 41].

However, it is imperative to differentiate the underlying mechanisms through which agricultural interventions impact these outcomes across the two settings. Complex humanitarian emergencies are the main driver of food and nutritional insecurity [6]. Thus, the latent factors which affect the impact pathways and outcomes of agricultural interventions in CHES are also likely to be impacted by violent conflict itself. CHES-driven factors such as restriction to access land and water resources, loss of productive and livestock assets, agricultural, crop damages, and agricultural labor shortages driven by displacement of people from rural areas are essential determinants of these nutrition and welfare outcomes [4]. In addition, CHES limit access to output and value chain markets for selling agricultural produce, which limits income-generation, availability, and supply of fresh produce in markets [4]. Agricultural intervention in CHES, hence, are theorized to improve auto-consumption of livestock and crop produce but not local production and consumption. Conflict could also lead to poor child nutrition through the lack of accessibility, availability, and affordability of healthcare facilities, and access to healthcare was not accounted for in any of the studies that assessed child nutrition outcomes. Apart from Vallet et al., [39] who investigated the role of rural markets, the role of contextual factors and the potential mechanisms of action in CHES were insufficiently explored. In addition, exposure to conflict directly shapes decision-making and risk-taking [42–43]. Displacement and population movement caused by conflict decreases farmer’s ability and willingness to invest in agriculture and can influence household participation and uptake of these interventions, as well as how they benefit from it. These factors are not prevalent in non-CHES.

As a result, this review was not able to determine specificities of the impact pathways linking agricultural intervention to nutrition, food security, and health in CHES. Therefore, these mechanisms and their implications on outcomes along the causal pathway in CHES need to be better investigated in future studies.

Finally, the low number of rigorous studies in CHES could be explained by two factors: (1) the lack of funding towards agricultural interventions in CHES and (2) the scarcity of good quality data in these settings.

First, development funding timelines and objectives differ substantially from humanitarian funding which tends to focus on responding to immediate and acute relief rather than building long-term resilience. Therefore, funding allocations to agriculture in CHES make up a fraction of that allocated to development programs and their evaluation [44].

The lack of studies and data emanating from CHES may also result from the reluctance of participants to accurately report production, consumption, and income in challenging settings. For example, respondents may under-report due to fear of losing assistance or no longer qualifying to receive it [45]. Another explanation that could apply to both types of settings, but is more accentuated in CHES, is the decrease in sample size mainly caused by attrition and access difficulties in the field, which could have prevented the identification of any effect. And although we find a similar lack of impact as previous reviews with respect to child stunting and underweight, reasons identified by authors are different, and include measurement bias and the inability to correctly estimate children’s dates of birth which are essential for the accuracy of anthropometric status indicators. Also, the constant movement of households, particularly of older children, who are often relocated to live with relatives can further decrease the sample of children available for follow up during surveys. It is also likely that in both types of settings, follow-up durations are not sufficient to identify an impact on anthropometric indices [34, 36]. Impact evaluation studies are also challenged by a myriad of methodological, ethical, and practical challenges, especially in CHES [46, 47]. Our review identified selection bias, spillover effect, attrition bias, information, recall, measurement biases, and non-random response as threats to internal validity. Moreover, many studies reported that data collected in such settings face logistical and practical challenges, which not only reduced sample sizes and underpowered the studies to identify any effect but also limited the study’s ability to measure, through process evaluation and intervention mapping, the implementation fidelity and the extent to which the impact could be attributed to the intervention itself [35–38, 48].

It is possible and feasible to use and adapt existing tested methods implemented in research studies from stable developing settings, including the use of RCTs, yet there is a need for exploring novel approaches to conducting impact evaluation in complex humanitarian emergency settings, which address some of the contextual ethical and practical challenges [46–47, 49]. In contexts where traditional face-to-face household surveys are difficult to conduct, alternative remote-based tools such online or mobile surveys [50], crowdsourcing [51–52], geospatial data [53], satellite data, and remote sensing [54] can be used to measure and assess outcomes (e.g. plot or land area, land and water use, crop production and productivity and market access). In addition, various studies included in this review have underscored attrition rates, potentially leading to smaller sample size at follow-up. To overcome this common challenge, impact evaluations in these types of contexts could consider oversampling techniques to prevent loss of statistical power and maintain the robustness of research findings. The included studies could also be strengthened by adopting mixed-method approaches, particularly involving stakeholders, which can provide a more complete understanding of the complex nature of resulting behaviors, experiences, differential impacts, and potential unintended consequences.

To our knowledge, this systematic review is the first to explore the potential impact of agricultural interventions on food security and nutrition outcomes in CHES. Our analysis focused exclusively on studies that compared outcomes between different groups, or before and after the intervention within the same group, which strengthened our findings, compared to studies with no control or comparator group. However, this study is subject to several limitations. The review was limited to studies written in English as the inclusion criteria, which could have excluded relevant studies in other languages. Furthermore, the interventions of the included studies were carried out in three countries (DRC, Northeast Nigeria, and Sudan), which limited the generalizability of the review to other countries and regions. From the articles identified, four were subjected to moderate risk of bias while two were deemed to be at a serious risk of bias, lowering the certainty of evidence of the impact of agricultural interventions on food security and nutrition outcomes in CHES. Finally, considering the aforementioned limitations and the nature of the studies included, we were not able to conduct a meta-analysis.

## Conclusion

Despite growing evidence on agriculture-nutrition linkages in low- and middle-income contexts, this review found little evidence of the impacts of agricultural intervention on food security and nutrition in complex humanitarian emergency settings, and the little evidence found offered a mixed picture. If agricultural interventions are to be considered as part of the toolbox to improve food security in these challenging settings, many more rigorous studies fulfilling this clear gap on the effectiveness of such interventions along their impact pathways are needed.

Particularly, there is a need for research from a range of geographical contexts and CHES intensities. This is crucial to fill the knowledge gaps on the role of agricultural and horticultural interventions on production, marketing, food consumption, nutrition, and child health. Contextual factors such as access to and availability of markets, land and water and healthcare services should be incorporated in the impact assessment as they are likely to moderate how agricultural interventions impact food security and nutrition.

## Data Availability

No datasets were generated or analysed during the current study.

## Abbreviations

BBC: Behavior Change Communication
CCES: Conflict and Complex Emergency Settings
DD: Dietary Diversity
DRC: Democratic Republic of Congo
F2F: Farmer-To-Farmer
FAO: Food and Agriculture Organization
FCS: Food Consumption Score
FFS: Farmers Field School
FGDs: Focus Group Discussions
HDDS: Household Dietary Diversity Score
HH: Household
HFIAS: Household Food Insecurity Access Scale
IDPs: Internally Displaced Populations
KIIs: Key Informant Interviews
LMICs: Low- and Middle-Income Countries
NGOs: Non-Governmental Organizations
PM2A: Prevention of Malnutrition in Children under Two Approaches
PRISMA: Preferred Reporting Items for Systematic Reviews and Meta-Analysis
RCSI: Reduced Coping Strategy Index
ROBINS-I: The Cochrane Risk of Bias In Non-randomized Studies of Interventions
SDG2: Sustainable Development 2
UNHCR: United Nations High Commissioner for Refugees
VSLA: Village Saving and Loan Associations
WEG: Women’s Empowerment Groups
